# Long-Term Effects of Polychlorinated Biphenyls and Dioxins on Pregnancy Outcomes in Women Affected by the Yusho Incident

**DOI:** 10.1289/ehp.10686

**Published:** 2008-02-06

**Authors:** Kiyomi Tsukimori, Shoji Tokunaga, Satoko Shibata, Hiroshi Uchi, Daisuke Nakayama, Tadayuki Ishimaru, Hitoo Nakano, Norio Wake, Takesumi Yoshimura, Masutaka Furue

**Affiliations:** 1 Department of Obstetrics and Gynecology; 2 Department of Preventive Medicine and; 3 Department of Dermatology, Graduate School of Medical Sciences, Kyushu University, Fukuoka, Japan; 4 Department of Obstetrics and Gynecology, Nagasaki University Graduate School of Biomedical Sciences, Nagasaki, Japan; 5 Fukuoka Institute of Health and Environmental Science, Fukuoka, Japan

**Keywords:** dioxin, environmental exposure, pregnancy loss, preterm delivery, spontaneous abortion, Yusho

## Abstract

**Background:**

Maternal exposure to polychlorinated biphenyls (PCBs) is associated with increased proportions of spontaneous abortion and stillbirth in animal studies. In Japan in 1968, accidental human exposure to rice oil contaminated with PCBs and other dioxin-related compounds, such as polychlorinated dibenzofurans (PCDFs), led to the development of what was later referred to as Yusho oil disease.

**Objective:**

The aim of this study was to investigate the association of maternal PCB and dioxin exposure with adverse pregnancy outcomes in Yusho women.

**Methods:**

In 2004, we interviewed 214 Yusho women (512 pregnancies) about their pregnancy outcomes over the past 36 years. Pregnancy outcomes included induced abortion, spontaneous abortion, preterm delivery, and pregnancy loss.

**Results:**

In pregnancy years 1968–1977 (within the first 10 years after exposure), the proportions of induced abortion [odds ratio adjusted for age at delivery (OR_adj_) = 5.93; 95% confidence interval (CI), 2.21–15.91; two-tailed *p* < 0.001) and preterm delivery (OR_adj_ = 5.70; 95% CI, 1.17–27.79; *p* = 0.03) were significantly increased compared with the proportions in pregnancy years 1958–1967 (10 years before the incident). Spontaneous abortion (OR_adj_ = 2.09; 95% CI, 0.84–5.18), and pregnancy loss (OR_adj_ = 2.11; 95% CI, 0.92–4.87) were more frequent (OR = 2.18; 95% CI, 1.02–4.66), but these were not significant (*p* = 0.11 and *p* = 0.08, respectively) in pregnancy years 1968–1977. We found no significant increases in the proportions of these adverse pregnancy outcomes in pregnancies occurring during 1978–1987 or 1988–2003 compared with those in pregnancies before 1968.

**Conclusion:**

High levels of PCB/PCDF exposure had some adverse effects on pregnancy outcome in Yusho women.

Polychlorinated biphenyls (PCBs), a group of persistent organochlorine compounds, are man-made chemicals first introduced in the late 1920s. In the late 1960s to the 1970s, many countries realized the potential dangers of PCBs and either banned them or restricted their use. However, PCBs are still present in the environment and in food chains because of their high resistance to abiotic and biotic degradation (Gladen et al. 1999; Wicklund-Glynn et al. 2000).

Maternal exposure to PCBs may affect pregnancy outcomes. Dietary PCB exposure increases the rate of spontaneous abortion and stillbirth in animals (Arnold et al. 1990; Barsotti et al. 1976; McNulty 1985). Only a few epidemiologic studies have examined the association between maternal exposure to PCBs and dioxins, and pregnancy outcomes in humans. A case–control study of Taiwanese women who consumed rice oil contaminated with PCBs and dioxins in the Yu-cheng incident showed an increase in stillbirth but not in spontaneous abortion (Yu et al. 2000). Higher proportions of spontaneous abortion and preterm delivery occurred in women who lived in a town contaminated with dioxin from a chemical plant in Chapayevsk, Russia (Revich et al. 2001). Increased maternal consumption of fish contaminated with dioxins and furans in Sweden was associated with lower infant birth weights (Rylander et al. 2000). However, recent cohort studies failed to support an association between maternal serum levels of chemicals and adverse pregnancy outcomes in women exposed to PCBs and dioxins (Axmon et al. 2004; Eskenazi et al. 2003; Sugiura-Ogasawara et al. 2003). Thus, it is controversial whether maternal exposure to PCBs and dioxins is associated with adverse pregnancy outcomes in humans. Although there are only a few studies evaluating groups with high levels of exposure, these studies are particularly informative with regard to the association between PCB and dioxin consumption and pregnancy outcomes.

In Western Japan in 1968, accidental human exposure to rice oil contaminated with PCBs and other dioxin-related compounds led to the development of what was later referred to as Yusho oil disease (Kunita et al. 1985). PCB contamination occurred in the production of cooking oil, during which commercial PCB preparations were used for heat exchange. Pyrolysis of PCBs and chlorinated benzenes at high temperatures produced polychlorinated dibenzofurans (PCDFs) and polychlorinated dibenzo-*p*-dioxins (PCDDs). Consequently, Yusho oil disease is now recognized as mixed toxicity from PCBs and dioxin-related compounds (Furue et al. 2005). Over 1,900 people presented with clinical symptoms, including acneform eruptions; pigmentation of the skin, nails, and conjunctivae; increased discharge from the eyes; and paresthesias of the extremities (Ikeda 1996; Urabe and Asahi 1985). More than 500 victims died thereafter. From an epidemiologic survey of 141 Yusho patients, Hayabuchi et al. (1979) estimated that the total amounts of PCBs, PCDFs, and polychlorinated quarterphenyls (PCQs) ingested by each patient were 633, 3.4, and 0.62 mg on average, respectively. From the follow-up data of 5 Yusho patients, fat-based concentrations of the toxic equivalent quantity (TEQ) and PCBs were estimated to have decreased from 40 ppb and 75 ppm, respectively, in 1969, to 0.6 ppb and 2.3 ppm, respectively, in 1999 (Masuda 2001, 2005). However, a recent study showed that from 2001 to 2003, mean blood levels of total dioxins (pg-TEQ/g lipid) and 2,3,4,7,8-penta-chlorodibenzofuran (PeCDF; picograms per gram lipid) in Yusho patients were 3.4–4.8 and 11.6–16.8 times higher, respectively, than the mean levels in normal controls (Furue et al. 2005). Thus, dioxins and PCBs persisted for a long time in Yusho patients.

The aim of this study was to investigate the relationship between maternal PCB or dioxin exposure and adverse pregnancy outcomes in Yusho women. We interviewed the living Yusho women about their pregnancy outcomes over the past 36 years. We also analyzed the relationship between blood levels of PCBs and dioxins, and adverse pregnancy outcomes.

## Materials and Methods

### Subjects

The nationwide health examination for Yusho has been conducted annually since 1986. Its purpose is to provide health maintenance services and to monitor the health status of chronic Yusho patients (Hirota et al. 1996). The examination is open not only to officially registered Yusho patients but also to those who regard themselves as potential victims. Participation in the examination is voluntary. From 1968 to 2003, the Study Group for Yusho registered 602 women according to the diagnostic criteria for Yusho based on signs and symptoms of the illness, a history of consumption of the contaminated oil, or the composition and concentration of blood PCBs and PCQs (Furue et al. 2005). Data on pregnancy outcomes were obtained either by face-to-face interviews (*n* = 36, 16.8%), by completion of mailed questionnaires (*n* = 125, 58.4%), or telephone interviews (*n* = 53, 24.8%) of each subject or a close relative. Interviews were conducted between April 2004 and December 2004. The interviewers were trained nurses who described the study as a health survey conducted by the Study Group for Yusho. The interviewers were not informed of the hypotheses concerning pregnancy outcomes. The questions about pregnancy outcomes addressed the number of pregnancies, number of deliveries, delivery dates, gestational ages at delivery, and birth weights; offspring outcomes included birth date, sex, life or death, and age at the interview. All interviewers were female. We obtained oral or written informed consent from all subjects prior to the interview and blood draw. The Institutional Ethics Committee approved the study design.

Of 602 officially registered Yusho patients, 119 had no traceable address, 129 did not return the questionnaire, and 18 declined to participate in this study. Of the remaining 336 subjects, 14 were offspring of exposed subjects, 3 had mental impairment (2 senile dementia, 1 mental retardation), 1 omitted her birth year and date, 10 gave no information on the number of pregnancies and/or children, 6 gave incomplete information on the date of pregnancy and/or child birth, and 5 gave contradictory pregnancy and delivery information (the birth year of a child was inconsistent with the age, for example). Of the remaining 297, 258 were answered by the patient herself and 29 were answered by patients’ mothers. The remaining 9 observations were provided by other surrogates (4 fathers, 2 sisters, 1 sister-in-law, 2 daughters), and 1 was provided by an undetermined responder. These 10 were excluded. Of the 287 women included, 30 were never pregnant, and the number of pregnancies was not known for 8 women. We excluded 33 women who completed their pregnancies 10 years before the Yusho incident and 2 who did not report the year of delivery; 214 subjects with pregnancies occurring within 10 years before or after the Yusho incident were eligible for the analysis. These 214 subjects had a total of 512 pregnancies during the corresponding period. We divided these 512 pregnancies into four groups according to the year of pregnancy: 1958–1967, 1968–1977, 1978–1987, and 1988–2003. For an additional analysis on pregnancy outcome relative to timing of the incident, we selected 42 women who had a total of 142 pregnancy outcomes in both the 1958–1967 period and the period after the Yusho incident. To assess the association of pregnancy outcomes with PeCDF and PCB exposure from the Yusho incident, we used outcomes from pregnancies 10 years before the Yusho incident (pregnancy years 1958–1967) as reference values.

### Pregnancy outcome measures

We assessed four pregnancy outcomes in this analysis: *a*) spontaneous abortion (pregnancy loss occurring < 22 weeks after the onset of the last menstrual period); *b*) preterm birth (birth at < 37 completed weeks of gestation); *c*) pregnancy loss (including spontaneous abortion, stillbirth, and neonates who could not be confirmed as being alive at birth); and *d*) induced abortion (medical or surgical termination of a pregnancy < 22 weeks after the onset of the last menstrual period).

### Measurement of PCBs and dioxin-related compounds

The serum dioxin-related compound levels were measured as a part of the nationwide health examination of Yusho poisoning, as described above. The blood samples of participants who were willing to have their dioxins levels measured were collected beginning in 2001. In the present study, we analyzed the measurements from blood samples taken during 2001–2005. We obtained serum dioxin-related compound levels of 97 women of the 214 eligible subjects. Most subjects were measured more than once. The numbers of measurements (number of subjects) were as follows: 1 (24), 2 (16), 3 (12), 4 (26), and 5 (19). Out of the 97 women, 52 women provided 105 pregnancy outcomes after the Yusho incident.

Among the 21 PCB and dioxin-related compounds measured, PeCDF, 3,3′,4,4′,5-pentachlorobiphenyl (PCB-126), and 3,3′,4,4′,5,5′-hexachlorobiphenyl (PCB-169) were selected for the statistical analyses because of their medical importance. Of the total toxicity in the adipose tissue and in the blood, 76% and 65%, respectively, were attributable to PeCDF retained in Yusho patients. In the serum of controls from the general population, PCB-126 was the main contributor to the total toxicity (23%), surpassing the contributions by other congeners of PCDDs, PCDFs, and PCBs (Masuda 2005). PCB-169, which is highly toxic, is the most abundant coplanar PCB in the blood of Yusho patients (Tokunaga et al. 2005). We measured blood PCB concentrations with a high-resolution gas chromatograph and a high-resolution mass spectrometer equipped with a solvent-cut large volume injection system as described elsewhere (Iida and Todaka 2003). An accelerated solvent extraction method was used for the treatment. The measurements of PCBs and dioxins were highly reproducible (Tokunaga et al. 2005). For PeCDF, PCB-126, and PCB-169, the differences in their geometric means, expressed as a percent [95% confidence interval (CI)] were 1.1% (–6.8 to 9.7), 12.2% (4.1 to 20.9), and 6.8% (–0.3 to 14.4), respectively; their intraclass correlation coefficients between the measurements of 2001 and 2002 were 0.98, 0.85, and 0.94, respectively.

The concentration of the chemical at the time of pregnancy was estimated as the concentration at sampling × 2^[duration between date of pregnancy and date of sampling (years)/half-life of chemical (years)]^. This equation was based on a simple decay model deduced from a single compartment model with a constant elimination rate (Weisskopf et al. 2003). The biologic half-lives of PeCDF, PCB-126, and PCB-169 were estimated to be 7.7, 14.6, and 14.6 years, respectively (Masuda 2005). In the event that more than one measurement was taken for a subject, we estimated the concentration at the time of pregnancy for each measurement. The geometric mean of the retrograde, extrapolated value was used as an estimate of the concentration of the compound at the time of the pregnancy. The blood lipid levels of the congeners before the Yusho outbreak were set at the geometric mean for the general population of the Fukuoka Prefecture, Japan (Masuda et al. 2005).

### Statistical analyses

We analyzed the associations between maternal exposure in the Yusho incident and pregnancy outcomes using a random effects logistic model, because a woman could have more than one pregnancy. To relax the assumption of independence among the responses of the same subject given the covariates, a subject-specific random intercept (i.e. the baseline risk) was included in the model. The likelihood function was calculated by adaptive Gauss-Hermite quadrature on the estimation of the parameters (Rabe-Hesketh and Skrondal 2005). The explanatory variables were dummy variables for birth date (categorized into yearly ranges: 1958–1967–1968–1977–1978–1987, and 1988–2003) and age at delivery as a continuous variable. The square of the age at delivery was also used to consider the possible curvature of the dose-log (odds) relationship. For the women who had pregnancies both before and after the Yusho incident, the association between the pregnancy outcomes before and after the Yusho incident were analyzed separately using a random effects logistic model. Blood lipid levels of PeCDF, PCB-126, and PCB-169 were log-transformed with a base of 10 because of their highly skewed distribution. The associations between these chemicals and pregnancy outcomes were analyzed using a random effects logistic model that was adjusted for age at delivery as a continuous variable. The square of the age at delivery was statistically significant in some combinations of the dependent variable and the explanatory variable, suggesting a curvature of the dose-log (odds) relationship. All tests were two-tailed, and *p*-values < 0.05 were considered statistically significant. All statistical analyses were performed with Stata/SE Statistical Software, release 9.2 (Stata Corporation, College Station, TX, USA).

## Results

Demographic and clinical characteristics of the study subjects are shown in Table 1. The 214 women had a total of 512 pregnancies during the study period. There were 204 pregnancies completed before exposure and 308 pregnancies after exposure. The mean numbers ± SDs of pregnancies and offspring were 2.8 ± 1.3 and 2.3 ± 1.0, respectively. The mean ± SD of the maternal age at pregnancy was 27.8 ± 4.4 years.

Pregnancy outcomes of both unexposed and exposed women are shown in Table 2. Before the exposure, 11 pregnancies (5.4%) ended as induced abortions. The proportions of spontaneous abortions, preterm delivery, and neonates whose viability at birth could not be confirmed were 7.3%, 0.6%, and 1.1% respectively. After the exposure, 21 (17.2%) pregnancies during 1968–1977 ended in induced abortions, and the proportions of spontaneous abortions, preterm deliveries, and neonates whose viability at birth could not be confirmed were 13.9%, 4.6%, and 2.3%, respectively. In contrast, the proportions of these adverse pregnancy outcomes in pregnancies during 1978–1987 and 1988–2003 were similar to those in unexposed women.

When we compared pregnancy outcomes after the Yusho outbreak with those before the incident, we found that the proportion of induced abortions statistically significantly increased in pregnancy years 1968–1977 [odds ratio adjusted for age at delivery (OR_adj_) 5.93, 95% CI, 2.21–15.91; *p* < 0.01] (Table 3). Spontaneous abortion was more frequent (OR_adj_ = 2.09; 95% CI, 0.84–5.18) in pregnancy years 1968–1977, but this was not significant (*p* = 0.11). The proportion of preterm deliveries was significantly increased in pregnancy years 1968–1977 (OR_adj_ = 5.70; 95% CI, 1.17–27.79; *p* = 0.03). Although we observed an increase in the proportion of pregnancy losses in pregnancy years 1968–1977 (OR_adj_ = 2.11; 95% CI, 0.92–4.87), the statistical significance was marginal (*p* = 0.08). There were no significant differences in the proportions of these adverse pregnancy outcomes between pregnancies before exposure and pregnancies after exposure during the periods 1978–1987 or 1988–2003.

The additional analysis on the women who had pregnancy outcomes both before and after the Yusho incident yielded similar results. Based on the date of delivery after the Yusho incident, with the date of delivery before the incident as a referent, the ORs were 8.53 (95% CI, 2.27–32.04; *p* < 0.01) for induced abortion among all pregnancies, 4.46 (95% CI, 1.45–13.75; *p* = 0.01) for spontaneous abortion among pregnancies except induced abortion, 2.88 (95% CI, 0.37–22.22; *p* = 0.20) for preterm delivery among all deliveries, and 4.92 (95% CI, 1.68–14.42; *p* < 0.01) for pregnancy loss among pregnancies except induced abortions. ORs were not adjusted because of intraperson analysis.

The estimated concentrations of PeCDF, PCB-126, and PCB-169 are shown in Table 4. In pregnancies during 1968–1977, the mean (range) concentrations of PeCDF, PCB-126, and PCB-169 were 2899.3 (112.1–19942.7), 336.4 (89.5–1705.3), and 759.6 (67.6–4224.2), respectively. The concentrations of PeCDF, PCB-126, and PCB-169 gradually decreased each year, but the mean concentrations of PeCDF, PCB-126, and PCB-169 in pregnancies during 1988–2003 were about 2- to 5-fold greater than those of the general population. Associations of pregnancy outcomes with the estimated concentrations of PeCDF, PCB-126, and PCB-169 are shown in Table 5. We found a statistically significant association between the proportion of induced abortions and the concentrations of PeCDF (OR adjusted for age at delivery (OR_adj_) = 1.82 (95% CI, 1.21–2.74; *p* < 0.01), PCB-126 (OR_adj_ = 4.14, 95% CI, 1.30–13.19; *p* = 0.02), and PCB-169 (OR_adj_ = 3.47, 95% CI, 1.587–7.61; *p* < 0.01). Statistically significant associations between other adverse pregnancy outcomes and the estimated concentrations of PeCDF and PCB-169 were also observed. The associations of the estimated concentration of PCB-126 with the proportions of spontaneous abortions and preterm deliveries were marginally significant.

## Discussion

In this retrospective survey, Yusho women exposed to high doses of PCBs and dioxins had a 2-fold increase in the proportion of spontaneous abortion, a 5-fold increased proportion of preterm delivery, a 2-fold increase in pregnancy loss, and a 5-fold increase in induced abortion. These differences were present in the 10 years immediately after the incident compared with the 10 years before. The association diminished over time, as differences in pregnancy outcomes were no longer present 10 years after the incident.

Epidemiologic studies examining the relationship between maternal exposure to PCBs/dioxins and pregnancy outcomes have demonstrated higher proportions of adverse outcomes including spontaneous abortion, stillbirth and preterm delivery, fetal growth restriction, and low birth weight (Guo et al.1995; Revich et al. 2001; Yu et al. 2000). The effects were not uniform across all of the studies. For example, women involved in Taiwan’s Yucheng incident had a higher proportion of stillbirth but not spontaneous abortion. Because all of these studies were observational studies, variability in the observations was not surprising given the array of possible confounding factors. The discrepancies in the results could be due to different levels of exposure and variations in the mixtures of toxicant compounds in each setting. Still, we too observed increases in spontaneous abortion, stillbirth, and preterm delivery. Although we did not survey birth weight in the present study, another study of Yusho women did document low birth weight and fetal growth restriction (Yamashita and Hayashi 1985). Therefore, it is plausible that the increased proportion of preterm delivery seen in our study would correspond to an increase in low birth weight infants.

A few studies have used biologic measures of dioxin exposure. One such study (Eskenazi et al. 2003) was a retrospective cohort study of 510 women (888 total pregnancies) residing in Seveso, Italy, who were exposed to 2,3,7,8-tetrachlorodibenzo-*p*-dioxin (TCDD) following an explosion. Serum levels of TCDD were measured shortly after the explosion. Ninety-seven pregnancies (10.9%) ended as spontaneous abortions. The researchers found no association between TCDD levels and either spontaneous abortion (adjusted OR = 0.8), birth weight (adjusted beta= –4 g), or the frequency of small-for-gestational-age infants (adjusted OR = 1.2) in the postexplosion pregnancies (Eskenazi et al. 2003). In a study in Sweden, women with miscarriages had lower estimated past 2,2′,4,4′,5,5′-hexachlorobiphenyl (PCB-153) concentrations than women with live births (Axmon et al. 2004). In a Japanese case–control study comparing 45 patients with recurrent miscarriages with 30 healthy women, Sugiura-Ogasawara et al. (2003) found no differences in mean serum levels of PCBs, hexachlorobenzene (HCB), and 1,1′-dichloro-2,2′-bis(*p*-chlorophenyl) ethylene (DDE). In contrast, the present study demonstrated a significant relationship between adverse pregnancy outcomes such as spontaneous abortion, preterm delivery, and pregnancy loss, and the blood concentrations of PCBs and dioxins. In addition, the estimated blood levels of PCBs and dioxins during 1968–1977 were about 10-to 400-fold greater than those of the general population. These levels gradually decreased, and their fall coincided with the improvement in pregnancy outcomes seen beginning 10 years after the Yusho incident.

The effects of PCBs on reproduction have been studied in animals. Monkeys exposed to PCBs had increased proportions of spontaneous abortion and stillbirth (Barsotti et al. 1976). Although the reproductive toxicity of PCDFs has not been directly studied, their toxicity is generally between that of PCBs and the chemically similar PCDDs (Safe 1990). PCB congeners can be estrogenic, anti-estrogenic, androgenic, or antiandrogenic (Ulbrich and Stahlmann 2004). TCDD, the most potent PCDD, also interacts with the estrogen receptor, acting as both an agonist and an antagonist (Safe and Wormke 2003). It causes spontaneous abortion in cynomolgus macaques (Guo et al. 1999). The primary endocrine alterations associated with TCDD treatment are significant decreases in serum estradiol and bioactive chorionic gonadotropin concentrations (Guo et al. 1999). These endocrine perturbations could possibly lead to placental insufficiency, compromised embryo circulation, and subsequent spontaneous abortion. Alternatively, as demonstrated with a model using pregnant rat uteri, the PCB mixture Aroclor 1242 increases the frequency of uterine contractions. This effect is meditated by arachidonic acid, which is released through activation of phospolipase A_2_ (Bae et al. 1999). Increased uterine irritability may have contributed to the increased proportion of preterm delivery in the Yusho women.

We did not measure hormone levels in the Yusho women in the present study. These women did, however, have decreased levels of the urinary estrogens pregnanediol and pregnanetriol 2 years after the exposure (Kusuda 1971). This may explain the increased proportion of spontaneous abortion and preterm delivery seen in the 10 years following the incident.

The present study had several limitations inherent to many retrospective studies. First, the women were interviewed years after the exposure, and not all were studied. The 287 Yusho patients who participated in this survey represented only 48% of the 602 registered Yusho patients. Second, the women were recalling events that took place years before the interview. Finally, social and medical environments, as well as lifestyles, might have changed over the years. Thus, our results may have been affected by selection bias, recall bias, and uncontrolled confounding factors.

Our study supports an association between PCB and dioxin exposure and adverse pregnancy outcomes. Yusho women had increased proportions of spontaneous abortion, preterm delivery, and pregnancy loss in the first 10 years after exposure. There was a significant relationship between adverse pregnancy outcomes and the concentrations of PCBs and dioxins. These effects diminished over time. Further studies, including those with additional follow- up of the offspring of Yusho mothers, are needed to confirm our observations.

## Figures and Tables

**Table 1:** Characteristics of the subjects.

Variable	No. (%)
Subjects (*n* = 214)	
Residence	
Fukuoka	102 (47.7)
Nagasaki	56 (26.2)
Other	56 (26.2)
Age at interview (years)	59.4 ± 12.2^a^
38–49	67 (31.3)
50–59	35 (16.4)
60–69	64 (29.9)
70–82	48 (22.4)
Marital status	
Single	1 (0.5)
Married	209 (99.5)
No. of pregnancies	2.8 ± 1.3^a^
0	—
1	24 (11.2)
2	76 (35.5)
3	64 (29.9)
4–10	50 (23.4)
No. of offspring	2.3 ± 1.0^a^
0	6 (2.8)
1	26 (12.2)
2	114 (53.5)
3	46 (21.6)
4–6	21 (9.9)
Pregnancies (*n* = 512)^b^	
Date of pregnancy (year)	
1958–1967	204 (39.8)
1968–1977	122 (23.8)
1978–1987	88 (17.2)
1988–2003	98 (19.2)
Age at pregnancy (years)	27.8 ± 4.4^a^
17–24	115 (22.5)
25–34	358 (69.9)
35–44	39 (7.6)

a Mean ± SD.

b Two pregnancies with unknown outcomes were excluded.

**Table 2:** Pregnancy outcomes according to the date of pregnancy.

	Before exposure	After exposure		
Pregnancy outcomes	1958–1967	1968–1977	1978–1987	1988–2003
All pregnancies	204	122	88	98
Age at pregnancy [years (mean ± SD)]	26.9 ± 3.9	27.7 ± 4.9	26.9 ± 3.8	30.9 ± 4.0
Induced abortions/all pregnancies^a^	11/204 [5.4% (3.1–8.8)]	21/122 [17.2% (11.8–23.8)]	1/88 [1.1% (0.1–5.3)]	3/98 [3.1% (0.8–7.7)]
Spontaneous abortions/pregnancies except induced abortion^a^	14/193 [7.3% (4.4–11.1)]	14/101 [13.9% (8.6–20.8)]	6/87 [6.9% (3.0–13.2)]	10/95 [10.5% (5.8–17.2)]
Gestational age at delivery
Term deliveries/deliveries^a^	176/179 [98.3% (95.7–99.5)]	81/87 [93.1% (86.8–97.0)]	79/81 [97.5% (92.4–99.6)]	82/85 [96.5% (91.1–99.0)]
Preterm deliveries/deliveries^a^	1/179 [0.6% (0.0–2.6)]	4/87 [4.6% (1.6–10.2)]	1/81 [1.2% (0.1–5.7)]	3/85 [3.5% (1.0–8.9)]
Stillbirth and unconfirmed survival status^b^/deliveries^a^	2/179 [1.1% (0.2–3.5)]	2/87 [2.3% (0.4–7.1)]	1/81 [1.2% (0.1–5.7)]	0/85 [0.0% (0.0–3.5)]

a Number [% (95% CI)].

b Number of neonates whose survival status was not confirmed.

**Table 3:** ORs (95% CIs) for pregnancy outcomes by the date of pregnancy.

	Before exposure	After exposure		
Pregnancy outcome	1958–1967	1968–1977	1978–1987	1988–2003
Induced abortion among all pregnancies	1 (referent)	5.93 (2.21–15.91)	0.22 (0.02–1.90)	0.70 (0.16–3.08)
	—	*p* < 0.001	*p* = 0.17	*p* = 0.63
Spontaneous abortion among pregnancies except induced abortion	1 (referent)	2.09 (0.84–5.18)	1.00 (0.32–3.09)	1.22 (0.41–3.63)
	—	*p* = 0.11	*p* = 1.00	*p* = 0.72
Preterm delivery among all deliveries	1 (referent)	5.70 (1.17–27.79)	1.46 (0.20–10.49)	2.09 (0.33–13.20)
	—	*p* = 0.03	*p* = 0.70	*p* = 0.43
Pregnancy loss among pregnancies except induced abortions	1 (referent)	2.11 (0.92–4.87)	1.02 (0.37–2.84)	1.01 (0.37–2.78)
	—	*p* = 0.08	*p* = 0.97	*p* = 0.98

ORs were adjusted for age at delivery as a continuous variable and the square of the age at delivery. Two-sided *p-*values are shown.

**Table 4:** Estimated blood levels of PeCDF and PCBs (pg/g lipid) at delivery.

		Year of delivery		
Congener	General population (*n* = 152)	1968–1977 (*n* = 69)	1978–1987 (*n* = 21)	1988–2003 (*n* = 15)
PeCDF	7.25 (2.2–26.0)	2,899.3 (112.1–19942.7)	697.7 (52.4–2289.6)	39.5 (4.0–951.8)
PCB-126	41.6 (5.0–430.0)	336.4 (89.5–1705.3)	159.0 (94.7–330.5)	60.4 (16.9–118.8)
PCB-169	37.1 (5.0–160.0)	759.6 (67.6–4224.2)	386.2 (61.5–1041.5)	70.6 (11.8–499.1)

Geometric mean (minimum–maximum). Data for the general population are from Masuda (2005).

**Table 5:** Association of pregnancy outcomes with the estimated concentrations of PeCDF and PCBs 126 and 169.

Pregnancy outcome	PeCDF [OR (95% CI)^a^*p*-value^b^]	PCB-126 [OR (95% CI)^a^*p*-value^b^]	PCB-169 [OR (95% CI)^a^*p*-value^b^]
Induced abortion among all pregnancies	1.82 (1.21–2.74) < 0.01	4.14 (1.30–13.19) 0.02	3.47 (1.58–7.61) < 0.01
Spontaneous abortion among pregnancies except induced abortions	1.60 (1.10–2.33) 0.01	2.52 (0.92–6.87) 0.07	2.28 (1.09–4.75) 0.03
Preterm delivery among all deliveries	1.98 (1.03–3.80) 0.04	4.90 (0.93–25.75) 0.06	4.12 (1.19–14.30) 0.03
Pregnancy loss among pregnancies except induced abortions^c^	1.70 (1.18–2.46) < 0.01	2.99 (1.16–7.73) 0.02	2.68 (1.32–5.48) < 0.01

a OR for 10-fold increase in blood lipid level (95% CI) adjusted for age at delivery as a continuous variable, and the square of the age at delivery considering the possible curvature of the dose-log(odds) relationship.

b Two-sided *p*-value.

c Fetal loss, including spontaneous abortion, stillbirth, and neonates with unconfirmed survival status.
